# Supplementary material for: Counterfactual analysis of differential comorbidity risk factors in Alzheimer’s disease and related dementias
Source: PLOS Digit Health. 2022 Mar 15;1(3):e0000018. doi: 10.1371/journal.pdig.0000018 (PMC9931358; doi:10.1371/journal.pdig.0000018)
Supplement: S1 Text — A. Definition of ADRD in EHRs, B. Cohort matching methods, C. Disentangle the dependency among comorbidities and ADRD. (PDF) [file pdig.0000018.s001.pdf]

Supplementary Material 1

A. Definition of ADRD in EHRs

Accurate ADRD identification in EHRs is difficult because ADRD diagnosis codes can be erroneously reported. EHRs are billing-purpose records, and the ADRD diagnosis codes can be assigned incorrectly, particularly in underrepresented groups. There are several different definitions to detect ADRD onsets: i) having either diagnosis codes (dx) or medications (rx), ii) having both dx and rx, and iii) having both dx and imaging procedure.

Having either dx or rx. We used this definition in our analysis. This definition can detect the most ADRD cases but potentially overestimate the ADRD cases. Some identified ADRD cases may in fact not have ADRD.

Having both dx and rx. This definition detects ADRD cases by having both medication and diagnosis. However, there are ADRD patients who haven't started medication regimen yet. Particularly, it has been reported that there is a significant racial disparities in anti-dementia medication use - approximately 30% higher of anti-dementia drugs use among non-Hispanic Caucasians compared to other racial/ethnic groups after adjusting demographics, socioeconomic status, healthcare access and utilization, and comorbidities.[1]

Having both dx and imaging procedures. Patients who have received an ADRD diagnosis along with imaging tests are highly likely to be correctly assigned to the codes. The imaging tests can be identified by procedure codes such as head CT (procedure ID=12237, 4655, 12249, 12239, 12238,168103), MRI (procedure ID=3960, 12261, 12263,12255), PET (procedure ID=12963). We computed the ADRD prevalence rate for different racial groups (see Table below). The ADRD rate matches the global population in general, and AD+Imaging rate is distorted, potentially due to social determinants. This definition can underestimate the ADRD cases and have bias toward underrepresented minorities.

Table. Statistics of 2014 cohort stratified by race in Cerner.

Race/ethnicity	Active population	ADRD diagnosis codes	ADRD diagnosis codes (%)	ADRD diagnosis codes and imaging procedures	ADRD diagnosis codes and imaging procedures (%)
African American	20,258	3,886	19.18%	312	0.97%

Asian American	3,794	565	14.89%	102	1.87%
Caucasian	193,449	29,852	15.43%	2,941	1.10%
Hispanic	541	84	15.53%	2	0.23%

By comparing the three definitions, we chose the definition with the least risk in racial disparity, which is having either dx or rx.

B. Cohort matching methods

Structural equation to define ADRD risk in EHRs is given as

$$\text{ADRD} \sim \alpha_0 \cdot \text{race} + \alpha_1 \cdot \text{comorbidities with known risk} + \alpha_2 \cdot \text{comorbidities with unknown risk}$$
$$+ \alpha_3 \cdot \text{age when observation starts} + \alpha_4 \cdot \text{age when observation ends} + \alpha_5 \cdot \text{sex}.$$

We matched ADRD subjects and non-ADRD subjects based on age and sex for each racial group, thus $\text{ADRD} \perp \text{Age}$, $\text{ADRD} \perp \text{Sex}$ or $\alpha_3 = \alpha_4 = \alpha_5 = 0$(Matching 1 in Fig. 1c), where $\perp$refers to independence. We are interested in ADRD risk given each racial group (| refer to condition) :

$$\text{ADRD} \mid \text{race} \sim \hat{\alpha}_1 \cdot \text{comorbidities with known risk} \mid \text{race}$$
$$+ \hat{\alpha}_2 \cdot \text{comorbidities with unknown risk} \mid \text{race}$$

after omitting the adjusted age and sex given racial group. The comorbidities (with either known or unknown risk) incidence differs by race. Our focus is to identify the effect of comorbidities with unknown risk that disproportionately affects racial groups, because we already know the differential effect of comorbidities with known risk (e.g., hypertension increases ADRD risk more in African Americans compared Caucasians). So, we matched African Americans and Caucasians based on the comorbidities with known risk using propensity scores (Matching 2 in Fig. 1c). That is, we calculated the probability of being African American given the comorbidities using logistic regression and selected pairs of African Americans and Caucasians that had the similar probability. After the matching, we obtained

$$\text{ADRD} \mid \text{race} \sim \bar{a}_1 \cdot \text{comorbidities with known risk}$$

$+ \bar{a}_2$ ·comorbidities with unknown risk | race

because the comorbidities with known risk ⊥ Race by matching.

There are several methods to obtain matched cohorts (e.g., stratified matching, nearest neighbor, radius matching, kernel matching, Mahalanobis metric matching). None of the matching methods is superior to others. It is important to select right matching methods based on the variable distribution in the control set. The nearest matching with radius and caliper is a reasonable choice if the control data is large and asymmetrically distributed.[2,3] To obtain the right radius size, we used the nearest neighbors matching with incremental radius adjustment. That is, we started with a small radius and increased radius size until the difference of estimated probabilities (propensity scores) is within standard mean difference.[4] We discarded samples whose values are outside of the range defined by the caliper. We used *pymatch*, a publicly available package for cohort matching.[5]

C. Disentangle the dependency among comorbidities and ADRD

Causal structure learning is to identify a graph that best describes given data using a graphical model, where nodes represent variables and edges represent conditional dependencies between the nodes.[6,7]
using conditional independent tests.

The causal inference consists of two steps: searching a set of causal graphs (i.e., structure learning) and predicting the effects of a manipulation from the causal models. The causal model search can be very complicated as the number of possible DAGs grows super-exponentially with the number of nodes. A constraint-based search uses conditional independence from data to find d-separation, and PC is one of the most popular constraint-based algorithms with moderate accuracy.

Structure learning of a causal graph is to search a DAG that encodes conditional independencies from observational data (Fig. S1). The graph can be interpreted in this way: If two nodes for random variables x and y are d-separated by a set of nodes for random variables S, then the x and y are conditionally independent given the set of S. (i.e., $x \perp y \mid S$). We have to define v-structure as the subgraph $i \rightarrow j \leftarrow k$ on the nodes i, j and k where i and k are not adjacent. There are several approaches to find the causal structure. PC algorithm consists of two phases: i) find (undirected) skeleton and ii) find direction. In skeleton phase, PC starts with a complete undirected graph G_0. For all

pairs of nodes i and j, if nodes i and j are marginally independent at significance level α, the edge between them is deleted and a separation set $\hat{S}[i, j]$and $\hat{S}[j, i]$ is empty set $\{\}$. After finishing all marginal independence tests and deleting some edges, the new graph is denoted as G_1. Next step is to test conditional independence with adjacent nodes, given any single node in $adj(G_1, i)\backslash\{j\}$or $adj(G_1, j)\backslash\{i\}$, where $adj(G, i)$ denotes the set of nodes in graph G that are adjacent to node i. If there is any node k such that $i \perp j \mid k$, the edge between i and j is removed and node $k$is saved in separation set $\hat{S}[i, j]$. If all adjacent pairs have been tested given one adjacent node, a new graph is denoted as G_2. The algorithm continues in this way by increasing the size of the conditioning set step by step, i.e., $i \perp j \mid k_1, ..., k_q$, until all adjacency sets in the current graph are smaller than the size of the $\hat{S}[i, j]$. In orienting direction phase, we find orientation in each unshielded triplet $i - k - j$such that the pairs $(i, k)$and $(j, k)$are each adjacent in the skeleton but $(i, j)$are not in the triplet of nodes (i, j, k). The triplet $i - k - j$is oriented as $i \rightarrow k \leftarrow j$ if k is not in $\hat{S}[i, j]$.

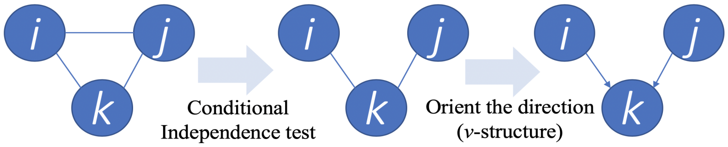

Figure S1. Finding directed acyclic graph for causal structure learning using PC algorithm. Find skeleton as testing for conditional independence with an increased cardinality of the conditioning set. Then propagate orientation as finding v-structure.

References

1. Zuckerman IH, Ryder PT, Simoni-Wastila L, et al. Racial and ethnic disparities in the treatment of dementia among Medicare beneficiaries. *J Gerontol B Psychol Sci Soc Sci*. 2008;63(5):S328-S333.

2. Baser O. Too much ado about propensity score models? Comparing methods of propensity score matching. *Value Health*. 2006;9(6):377-385.

3. Caliendo M, Kopeinig S. SOME PRACTICAL GUIDANCE FOR THE IMPLEMENTATION OF PROPENSITY SCORE MATCHING. *Journal of Economic Surveys*. 2008;22(1):31-72. doi:10.1111/j.1467-6419.2007.00527.x

4. Austin PC. Balance diagnostics for comparing the distribution of baseline covariates between treatment groups in propensity-score matched samples. *Stat Med*. 2009;28(25):3083-3107.

5. benmiroglio. benmiroglio/pymatch. Accessed May 18, 2021. https://github.com/benmiroglio/pymatch

6. Pearl J. *Causality: Models, Reasoning, and Inference*. Cambridge University Press; 2000.

7. Pearl J. *Probabilistic Reasoning in Intelligent Systems: Networks of Plausible Inference*. Elsevier; 2014.

8. Austin PC. An Introduction to Propensity Score Methods for Reducing the Effects of Confounding in Observational Studies. *Multivariate Behav Res*. 2011;46(3):399-424.
