# Supplementary material for: Counterfactual analysis of differential comorbidity risk factors in Alzheimer’s disease and related dementias
Source: PLOS Digit Health. 2022 Mar 15;1(3):e0000018. doi: 10.1371/journal.pdig.0000018 (PMC9931358; doi:10.1371/journal.pdig.0000018)
Supplement: S1 Table — Selected known comorbidities that we controlled to build a balanced cohort. (DOCX) [file pdig.0000018.s002.docx]

**Supplementary Table**

**Table S1**. Selected known comorbidities that we controlled to build balanced cohort

|  | ICD-9 | ICD-10 |
| --- | --- | --- |
| Hypertension | 401-405 | I10-I15 |
| Diabetes | 250 | E10-E14 |
| Obesity | 278.00, 278.01 | E66.* (except for E66.3*) |
| Heart disease | 390-398, 402, 404, 410-429 | I00-I09, I11, I13, I20-I51 |
| Vascular disease | 410.*, 427.31, 427.32, 428.*, 430, 431, 433.*1, 434.*1 | I10*, I48.91, I48.92, I50*, I60.9, I61.9, I63* |
| Head injury | 959.01, 850.*, 854.* | S06*, S04.*, S07.*, S09.* |
