# Supplementary material for: Counterfactual analysis of differential comorbidity risk factors in Alzheimer’s disease and related dementias
Source: PLOS Digit Health. 2022 Mar 15;1(3):e0000018. doi: 10.1371/journal.pdig.0000018 (PMC9931358; doi:10.1371/journal.pdig.0000018)
Supplement: S3 Table — Definition of late effect of cerebrovascular disease and transient cerebral ischemia. (DOCX) [file pdig.0000018.s004.docx]

**Supplementary Material 3**

**Definition of late effect of cerebrovascular disease**

**PheWas 433.8, ICD9 438.* Late effect of cerebrovascular disease**

| Late effects of cerebrovascular disease |
| --- |
| Cognitive deficits as late effect of cerebrovascular disease |
| Speech and language deficits as late effect of cerebrovascular disease |
| Speech and language deficit, unspecified, as late effect of cerebrovascular disease |
| Aphasia as late effect of cerebrovascular disease |
| Dysphasia as late effect of cerebrovascular disease |
| Late effects of cerebrovascular disease, dysarthria (ADDED) |
| Late effects of cerebrovascular disease, fluency disorder (ADDED) |
| Other speech and language deficits as late effect of cerebrovascular disease |
| Hemiplegia/hemiparesis as late effect of cerebrovascular disease |
| Hemiplegia affecting unspecified side as late effect of cerebrovascular disease |
| Hemiplegia affecting dominant side as late effect of cerebrovascular disease |
| Hemiplegia affecting nondominant side as late effect of cerebrovascular disease |
| Monoplegia of upper limb as late effect of cerebrovascular disease |
| Monoplegia of upper limb affecting unspecified side as late effect of cerebrovascular disease |
| Monoplegia of upper limb affecting dominant side as late effect of cerebrovascular disease |
| Monoplegia of upper limb affecting nondominant side as late effect of cerebrovascular disease |
| Monoplegia of lower limb as late effect of cerebrovascular disease |
| Monoplegia of lower limb affecting unspecified side as late effect of cerebrovascular disease |
| Monoplegia of lower limb affecting dominant side as late effect of cerebrovascular disease |
| Monoplegia of lower limb affecting nondominant side as late effect of cerebrovascular disease |
| Other paralytic syndrome as late effect of cerebrovascular disease |
| Other paralytic syndrome affecting unspecified side as late effect of cerebrovascular disease |
| Other paralytic syndrome affecting dominant side as late effect of cerebrovascular disease |
| Other paralytic syndrome affecting nondominant side as late effect of cerebrovascular disease |
| Other paralytic syndrome, bilateral |
| Alterations of sensations |
| Disturbances of vision |
| Other late effects of cerebrovascular disease |
| Apraxia as late effect of cerebrovascular disease |
| Dysphagia as late effect of cerebrovascular disease |
| Facial weakness |
| Ataxia as late effect of cerebrovascular disease |
| Vertigo as late effect of cerebrovascular disease |
| Other late effects of cerebrovascular disease |
| Unspecified late effects of cerebrovascular disease  **Definition of Transient cerebral ischemia**  **PheWas 433.31, ICD9 435.* Transient cerebral ischemia**  Transient cerebral ischemia  Basilar artery syndrome  Vertebral artery syndrome  Subclavian steal syndrome  Vertebrobasilar artery syndrome  Other specified transient cerebral ischemia  Unspecified transient cerebral ischemia |
