# Supplementary material for: Counterfactual analysis of differential comorbidity risk factors in Alzheimer’s disease and related dementias
Source: PLOS Digit Health. 2022 Mar 15;1(3):e0000018. doi: 10.1371/journal.pdig.0000018 (PMC9931358; doi:10.1371/journal.pdig.0000018)
Supplement: Attachment — Submitted filename: Response to Reviewer.pdf [file pdig.0000018.s005.pdf]

(PDIG-D-21-00037) Counterfactual Analysis of Differential Comorbidity Risk Factors in Alzheimer's Disease and Related Dementias

Response to Reviewers

We thank the reviewers for the constructive comments. We have significantly modified the manuscript to meet the reviewers' suggestions.

Reviewer 1

1-I wonder what the difference is between counterfactual analysis and adding the propensity score as a variable in the regression model.

➜ We thank Reviewer 1 for raising this question. Counterfactual analysis (or treatment effect estimation) is a general term to compare counterfactual outcomes in relation to factual outcomes, to pinpoint the causal relationship of one variable T to outcome Y. To estimate the treatment effect $\tau$of variable (or treatment) T, which is defined as the difference between factual and counterfactual outcomes, several methods have been proposed, such as propensity score matching/weighting or structural equation modeling. The structural equation model can be defined as $Y = \alpha + \tau \cdot T + \epsilon, T = f(X) + \eta$ (as a simple linear equation, for example) where $f(X)$denotes propensity scores. I believe Reviewer 1's "adding the propensity score as a variable in the regression model" refers to structural equation modeling, and it is one type of method in counterfactual analysis.

2- I wonder if there are any similar health care studies or examples using Counterfactual effects with Bayesian networks.

➜ We thank Reviewer 1 for the critical comments. We acknowledge that there were not enough literature surveys in the Introduction section. We have updated literature surveys in the Introduction section to provide readers with healthcare studies/examples using counterfactual analysis and Bayesian networks as follows:

"The statistical inferences to estimate the causality and counterfactuals from observational data in medicine have been long discussed but not yet widely used.[1–9] For example, previous research proposes a framework for emulating randomized trials from big observational data.[7,10,11] This framework simulates randomized clinical trials via controlling baseline characteristics, identifying time zero (baseline) to the outcome, adjusting the confounders by matching, and estimating treatment effect using potential outcome models.[10] A challenge here is that latent confounders can lead to selection bias; the causal structure can delineate the relationship between these confounders and help reduce the selection bias.[12] Indeed there is a separate line of causal analysis studies utilizing causal structure learning to investigate

conditional independence among comorbid conditions,[13–18] mainly with a few predetermined selected variables due to super-exponential complexity in structure learning.”

> 3- I am curious how to interpret the result of Average Treatment Effect(ATE). In the field of public health or medicine, it is important to identify the types of disease causes and measure the size of risk (i.e., odds ratio or hazard ratio). In particular, the interpretation of risk is very important. For example, smokers are four to five times more likely to develop lung cancer than non-smokers. Please describe how it is desirable to interpret ATE.

➙ Thanks for sharing this important question. We agree with Reviewer 1 that more details on ATE interpretation are needed. ATE means the difference between outcomes of the treated and outcomes of the untreated (for simplicity, let’s assume the two groups follow the same pretreatment conditions, like in randomized clinical trials). ATE=$\tau > 0$ means that the outcome when subjects are intervened to have the comorbidity is greater by τ than the outcomes when subjects are intervened not to have the comorbidity, implying the comorbidity exposure increases the outcomes on average. Similarly, ATT=$\tau > 0$ means that the outcome of subjects already having the comorbidity is greater by τ than the outcomes when the subjects are intervened not to have the comorbidity, implying the subjects with the comorbidity would have decreased the outcome (ADRD onset) by τ if they are without the comorbidity. For example, in the African American cohort, Its ATE (by IPTW[19]) was 0.2715 with 95% CI = [0.2423, 0.3137] (Table 3), which implies that having this comorbidity increases the risk for ADRD by 27.15% point on average. Similarly, the ATT of 0.1908 implies that African Americans with the late effect of CVD would have decreased the risk for ADRD by 19.08% point if their CVD was intervened. We have added the details in the Method and Results section.

Reviewer 2

> 1- In this study, a Causal Bayesian network was used to find unknown ADRD-inducing comorbidities. However, this study does not provide quantitative results that the proposed method is effective in finding ADRD-inducing comorbidities. To prove that the proposed model can detect ADRD-inducing comorbidities, it should be suggested to show how many of the known comorbidities are detected through the proposed model when the proposed model is analyzed without matching the known comorbidities. Then, reliability can be obtained even for the result of finding the unknown comorbidities.

➙ Thanks for Reviewer 2’s constructive suggestions. These are very critical points and we agree that quantifying how many of the known comorbidities are detected by the model without matching the known comorbidities between ADRD and non ADRD groups is a great idea to show the validity and reliability of the model.

We defined the known comorbidities as a few chronic diseases that have strong evidence to predispose subjects to ADRD. Actual high-risk comorbidity to ADRD is not limited to this selected set of chronic diseases. We did not match the known comorbidities in the ADRD and non-ADRD groups (we matched them in two racial groups, see Table 1). So the data fed into the Bayesian

network model contained the known comorbidities (defined as a chronic disease with strong evidence) and also other comorbidities, without matching by ADRD. And the Bayesian network found several edges from various comorbidities to ADRD. Some examples of edges are cerebrovascular disease (433.8) or mental disorders (296.2), see figure below. These two comorbidities have some supporting evidence on their risk predisposing older adults to ADRD, [20–22] showing the reliability of the Bayesian Network by confirming the existing knowledge. Our finding was that these comorbidities with medium-level evidence had disproportionate risk in the two racial groups.

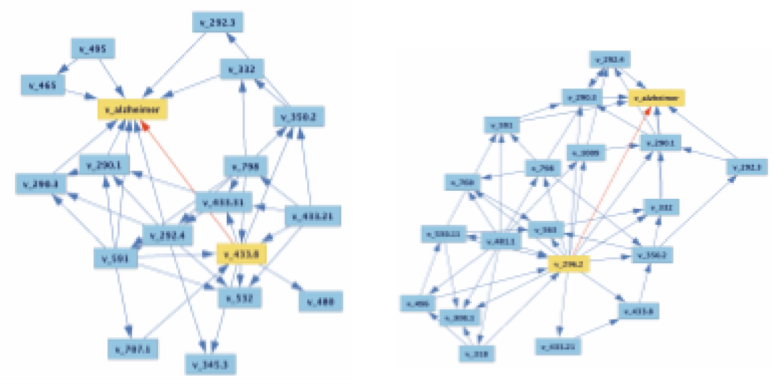

Figure. Bayesian Network without matching the known comorbidities between ADRD and non ADRD group.

2- The constructed Bayesian network itself could be a biased network for this study data. please show whether building a network multiple times with different subsamples has the similar result. Moreover, I hope you can prove that the networks between the two races are significantly different..

→ Thanks for Reviewer 2's suggestions. For each of the two racial groups (Caucasian and African American), we randomly split the dataset into 10 sub-datasets, each is a subset of the whole dataset with 10% data withhold. We run the PC algorithm 10 times on each of the 10 sub-datasets and aggregate the 10 causal graphs together. We count the number of appearances of each edge in the 10 graphs to reduce the bias. Below please see the number of the appearance of each edge related to "Alzheimer". This verifies our findings that 433.8 and 296.2 are two significant factors causing Alzheimer's in the different racial groups. We have added the robustness check by the ensemble in the Method Section (Identify comorbidities with potential causal effect) and Supplementary Material 2.

Table A: # Appearance of edges to "Alzheimer" in African American

Cause	Effect	# Appearance
Hypovolemia (276.5)	Alzheimer	7
Dementias (290.1)	Alzheimer	10

Other persistent mental disorders due to conditions classified elsewhere (290.3)	Alzheimer	10
Memory loss (292.3)	Alzheimer	10
Altered mental status (292.4)	Alzheimer	10
Parkinson's disease (332)	Alzheimer	10
Transient cerebral ischemia (433.31)	Alzheimer	7
Late effects of cerebrovascular disease (433.8)	Alzheimer	10
Acute upper respiratory infections of multiple or unspecified sites (465)	Alzheimer	10
Urinary tract infection (591)	Alzheimer	10
Fever of unknown origin (783)	Alzheimer	8
Essential hypertension (401.1)	Alzheimer	5
Syncope and collapse (788)	Alzheimer	3
Malaise and fatigue (798)	Alzheimer	1

Table B: # Appearance of edges to “Alzheimer” in the Caucasian counterpart

Cause	Effect	# Appearance
Dementias (290.1)	Alzheimer	10
Other persistent mental disorders due to conditions classified elsewhere (290.3)	Alzheimer	10
Memory loss (292.3)	Alzheimer	10
Altered mental status (292.4)	Alzheimer	10
Parkinson's disease (332)	Alzheimer	10
Depression (296.2)	Alzheimer	9
Urinary tract infection (591)	Alzheimer	5

To verify the causal graphs of the two racial groups are significantly different, for each node, we count how many neighbors in each of the two causal graphs, and check how many neighbors are common in the two graphs. In Supplementary Material 4. we provide the complete causal graphs of the two racial groups, as well as their comparison with each other as Venn diagrams for each node (variable). In the Venn diagram, the red circle shows its neighbors in the causal graph of the Caucasian group, and the green circle shows its neighbors in the causal graph of the African American group, to have an idea of the difference of the two causal graphs. We further evaluate the similarity of the two causal graphs by the structural hamming distance and get a result of 1,277, which means the number of edge insertions, deletions, or flips in order to transform one graph to another graph.

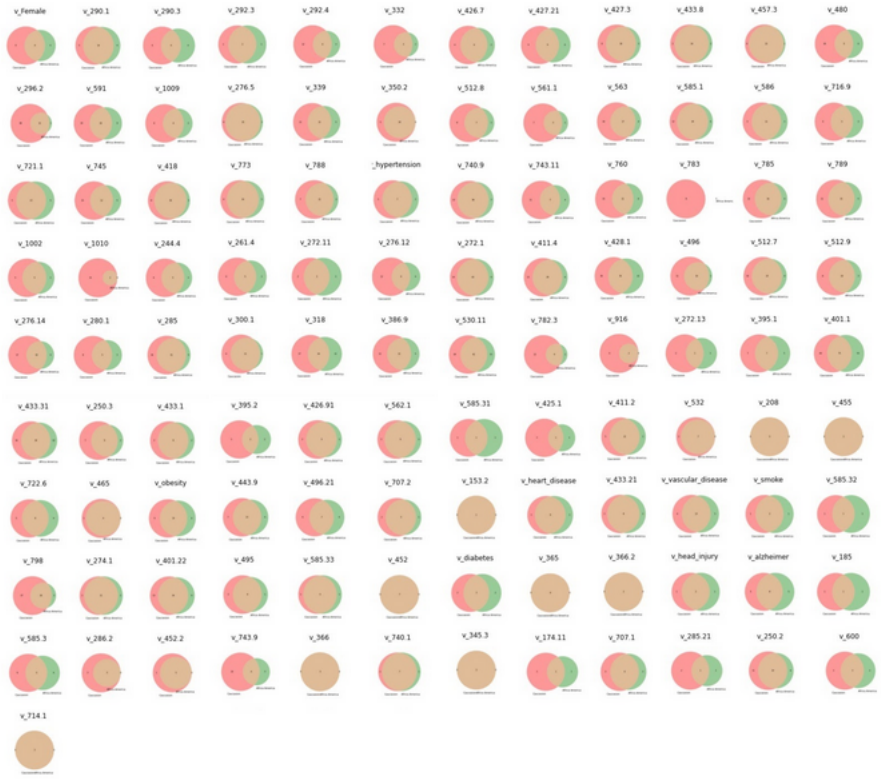

Figure S4.3. Comparison of the two causal graphs of the two racial groups. For each node, the red circle shows its neighbors in the Causasian causal graph, the green circle shows its neighbors in the African American causal graph, and the yellow area is the common neighbor nodes. The two causal graphs' structural hamming distance is 1,277.

3- Please provide the p-value whether the matched result statistically removed the difference significantly in Table 1.

➔ We thank Reviewer 2 for critical points. We agree with Reviewer 2 that we need to show whether the differences were removed statistically significant in the matching process. Following a prior work on evaluating the matching and assessing balance,[23] we calculated the standardized bias before and after the matching. The standardized bias of a variable is defined as the difference of means between the African American and Caucasian divided by the standard

deviation in African Americans. We thank Reviewer 2 for letting us know about the missing balance assessment. We have revised the manuscript accordingly in Table 1.

4- In Supplementary S2, please write ADRD's Markov blanket by name and code together for readability.

➜ We thank Reviewer 2 for pointing out the suggestions. We have revised Supplementary S2 accordingly.

Reviewer 3

1-Overall, there are several strengths to this study. The goal of the study is very timely, and the methods used are promising. In addition, authors utilized a large clinical database covering EHRs from more than 600 Cerner client hospitals which makes the results more interesting.

➜ Thanks for Reviewer 3's encouraging words.

2-The first major consideration is about the Bayesian network. To disentangle the multifactorial effect of comorbidities, the authors derived a Bayesian network of comorbidities and ADRD using constraint-based algorithms. Then the authors estimated the ATE of the selected comorbidities on ADRD. How to evaluate the results of the Bayesian network? Because a different method may result in different selected comorbidities.

➜ Thanks for Reviewer 3's critical comments. Evaluating the Bayesian network is indeed a very challenging task because the ground truth to compare does not exist. Our strategy to obtain a reliable Bayesian network relies on two techniques:

1. Add prior knowledge. We leverage prior knowledge by forbidding certain edges, such as diseases causing gender, factors of later time causing another factor of an earlier time, etc. Forbidden these types of edges to reduce the search space of the PC algorithm and help produce more accurate results with less bias.
2. Perform multiple runs with bagged data. We split the dataset into multiple (10) sub-datasets and run the causal inference algorithms on each dataset and aggregate the causal graphs. In the final aggregated causal graph, we count the number of appearances of each edge to evaluate the confidence of each edge. Edges appearing 10 times (in all 10 causal graphs) have higher reliability than edges that only appear once.

We have revised the manuscript to acknowledge the limitation of the Bayesian network. We also add Supplementary Material 4 to draw the difference between the two aggregated causal graphs of the two racial groups. For each node, we count how many neighbors are in each of the two causal graphs, and check how many neighbors are common in the two graphs. We provide the detailed figures as Venn diagrams (Supplementary material 4). For each node (variable), in the Venn diagram, the red circle shows its neighbors in the causal graph of the Caucasian group, and

the green circle shows its neighbors in the causal graph of African American group, to have an idea of the difference of the two causal graphs. We further evaluate the similarity of the two causal graphs by the structural hamming distance and get a result of 1,277, which means the number of edge insertions, deletions, or flips in order to transform one graph to another graph.

3-If the authors estimated the ATE of all the comorbidities, what will happen? Is it possible that some of the unselected comorbidities (by Bayesian network) may have higher/lower average treatment effect?

➙ Thanks for Reviewer 3's question. This is a very interesting idea. Reviewer 3's question can be rephrased as "what is the fundamental difference between the structural approach (e.g. Bayesian network) and non-parametric treatment effect approach (e.g., ATE estimation)". Indeed the structural and treatment effect approach are two competing paradigms of causal analysis.[24]

The structural approach articulates the underlying disease etiology between disease (comorbidities to ADRD) either from existing clinical knowledge or from learning the directed acyclic graphs (e.g., Bayesian Network).

The nonparametric treatment effect estimation focuses on a specific "treatment" when "treatment group" and "control group" exist. It is mainly for evaluating the impact of certain "treatments" (e.g., exposure to comorbid conditions, medications), rather than for forecasting new causal relationships.

The structural approach and the nonparametric treatment effect estimation are equivalent, under the linear structural model assumption.[25] ATE is$E[Y(1)] - E[Y(0)]$(treatment is randomized). This difference in average is the effect size obtained by a two-sample t-test. The two-sample t-test is equivalent to the simple ordinary least square in the linear model. Assume that our structural equation follows a linear model $Y = \alpha + \tau \cdot T + \epsilon$, where $\alpha$is an intercept, $T$is treatment assignment, and $\epsilon$is a random error. The ATE is then $(\alpha + \tau \cdot 1 + \epsilon) - (\alpha + \tau \cdot 0 + \epsilon) = \tau$. So estimating the edge (τ) in the structural model is equivalent to estimating ATE.

Now let's get back to our paper. We also assumed a linear model in the structural model. So, in theory, variables with statistically significant ATE would have edges in the structural model. In other words, "if we estimate the ATE of all the comorbidities..", only the "..selected comorbidities (by Bayesian network) may have (statistically significant) higher/lower average treatment effect".

Empirically, however, this claim and proof may not hold in our data, due to difficulty in estimating the true structure. So we speculate that edges from structural models and significant ATE may have overlap but are not equivalent.

4-The key elements of study design are unclear, e.g., observation period, following up period, index date. Please add further details and clarification.

→ Thanks for Reviewer 3's critical comments. We have added the study design details (including follow-up period) in the MATERIALS - Observation period section and Table 1.

MATERIALS - Observation period

"Age is the strongest risk factor for ADRD. Non-ADRD subjects were either i) not old enough to have ADRD onset (e.g., average ADRD onset age was 79.99 for African Americans and 81.57 for Caucasian), ii) old enough but right censored (e.g., median observation length was 1.0 years), or iii) old enough but free of ADRD. To avoid bias caused by different age distributions in ADRD and non-ADRD subjects, we matched age in their observation period (Fig. 1b, 1c Matching 1). That is, for the ADRD subjects, the observation window started from when any diagnosis code was first recorded and ended when the first ADRD onset was recorded (Fig. 1d). For the non-ADRD subjects, we selected subjects that had the closest age at the observation starts and ends. We truncated non-ADRD observations after the age when matched ADRD observations ended (Fig. 1d)."

Table 1. Summary of the study design based on Target Trial framework. We performed this analysis for African American and Caucasian counterparts, respectively.

**Protocol component**	**Description**
Eligibility criteria	Older adults with the age of 65 and older during the observation between 2000-2017. Observation length >6 months
Treatment strategies	Exposure to comorbidity. The comorbidities are selected based on the structural causal model (i.e.., Bayesian network)
Assignment procedures	To emulate random assignment of treatment, confounding variables were adjusted via propensity score matching, inverse weighting, or stratification. Confounding variables were identified based on structural causal model (i.e., Bayesian network)
Sampling strategies	100 bootstraps by random selection with replacement. 95% confidence interval reported
Follow-up period	Follow-up ends at ADRD onset (for ADRD subjects) and similar ADRD onset age (for non-ADRD subjects). Follow-up starts when the first comorbidities are recorded. The Median follow-up period was 1.0 years.
Outcome	ADRD onset after the comorbidities. ADRD onset identified by multiple PheWas codes and medication codes

Analysis plan	Compare the average number of ADRD onset between the comorbidity exposure group and non-exposure group

5- How does the study design deal with the censored patients?

➜ Thanks for Reviewer 3's constructive comments. Non-ADRD subjects were either not old enough to have ADRD onset (e.g., average ADRD onset age was 79.99 for African Americans and 81.57 for Caucasians) or old enough but censored. Case i) was not regarded as censored; they were excluded in the cohort. Case ii) is more concerning; censored older adults patients were very common in our data and the median observation period was 1.0 years (min 0.5 years, max 11 years). We have acknowledged our limitations in considering the short observation period and thus censored patients, as follows: "The median observation period was 1.0 years (min was 0.5 year, max 11 years), implying the non-ADRD subjects might be censored too early to observe their ADRD risk"

6-The authors used inverse probability of treatment weighting to estimate the counterfactual effect of comorbidities that have a direct edge to ADRD in each racial group's Bayesian network. To replicate the study and demonstrate the randomized emulation, the setting and results of each step of IPTW should be provided, e.g., unbalanced ratio, SMD threshold.

➜ Thanks to Reviewer 3 for raising the replication issue. To replicate the cohort matching, we have added SMD in Table 2 to show the balance after cohort matching between African American and Caucasian counterparts. To replicate the average treatment effect estimation, we provided a Jupyter Notebook that contains the codes and step-by-step configuration https://github.com/yejinjkim/treatment-effect.

Reference

1. Pearl J. Causality: Models, Reasoning, and Inference. Cambridge University Press; 2000.
2. Pearl J. Probabilistic Reasoning in Intelligent Systems: Networks of Plausible Inference. Elsevier; 2014.
3. James J. McGough SVF. Estimating the Size of Treatment Effects: Moving Beyond P Values. Psychiatry . 6: 21.
4. Ohlsson H, Kendler KS. Applying Causal Inference Methods in Psychiatric Epidemiology: A Review. JAMA Psychiatry. 2020;77: 637–644.
5. Parascandola M, Weed DL. Causation in epidemiology. J Epidemiol Community Health. 2001;55. doi:10.1136/jech.55.12.905
6. Zenil H, Kiani NA, Zea AA, Tegnér J. Causal deconvolution by algorithmic generative models. Nature Machine Intelligence. 2019;1: 58–66.
7. Prosperi M, Guo Y, Sperrin M, Koopman JS, Min JS, He X, et al. Causal inference and counterfactual prediction in machine learning for actionable healthcare. Nature Machine Intelligence. 2020;2: 369–375.
8. Glymour MM, Spiegelman D. Evaluating Public Health Interventions: 5. Causal Inference in Public Health Research-Do Sex, Race, and Biological Factors Cause Health Outcomes? Am J Public Health. 2017;107: 81–85.
9. Ling Y, Upadhyaya P, Chen L, Jiang X, Kim Y. Heterogeneous Treatment Effect Estimation using machine learning for Healthcare application: tutorial and benchmark. 2021. Available: http://arxiv.org/abs/2109.12769
10. Miguel A. Hernán JMR. Using Big Data to Emulate a Target Trial When a Randomized Trial Is Not Available. Am J Epidemiol. 2016;183: 758.
11. Chen Z, Zhang H, Guo Y, George TJ, Prosperi M, Hogan WR, et al. Exploring the feasibility of using real-world data from a large clinical data research network to simulate clinical trials of Alzheimer's disease. NPJ digital medicine. 2021;4. doi:10.1038/s41746-021-00452-1
12. Leite WL, Stapleton LM, Bettini EF. Propensity Score Analysis of Complex Survey Data with Structural Equation Modeling: A Tutorial with Mplus. Structural Equation Modeling: A Multidisciplinary Journal. 2019. pp. 448–469. doi:10.1080/10705511.2018.1522591
13. Kim Y, Jeong J-E, Cho H, Jung D-J, Kwak M, Rho MJ, et al. Personality Factors Predicting Smartphone Addiction Predisposition: Behavioral Inhibition and Activation Systems, Impulsivity, and Self-Control. PLoS One. 2016;11: e0159788.
14. Park YH, Kim Y, Yu H, Choi IY, Byun S-S, Kwak C, et al. Is lymphovascular invasion a powerful predictor for biochemical recurrence in pT3 N0 prostate cancer? Results from the K-CaP database. Sci Rep. 2016;6: 25419.
15. Faruqui SHA, Alaeddini A, Jaramillo CA, Potter JS, Pugh MJ. Mining patterns of comorbidity evolution in patients with multiple chronic conditions using unsupervised multi-level temporal

Bayesian network. PLoS One. 2018;13: e0199768.

16. McNally RJ, Mair P, Mugno BL, Riemann BC. Co-morbid obsessive-compulsive disorder and depression: a Bayesian network approach. Psychol Med. 2017;47. doi:10.1017/S0033291716003287

17. Upadhyaya P, Zhang K, Li C, Jiang X, Kim Y. Scalable Causal Structure Learning: New Opportunities in Biomedicine. 2021. Available: http://arxiv.org/abs/2110.07785

18. Wittmann L, Moergeli H, Martin-Soelch C, Znoj H, Schnyder U. Comorbidity in posttraumatic stress disorder: a structural equation modelling approach. Compr Psychiatry. 2008;49. doi:10.1016/j.comppsych.2008.02.004

19. Rosenbaum PR. Model-Based Direct Adjustment. Journal of the American Statistical Association. 1987. pp. 387–394. doi:10.1080/01621459.1987.10478441

20. Kokmen E, Beard CM, Chandra V, Offord KP, Schoenberg BS, Ballard DJ. Clinical risk factors for Alzheimer's disease: A population-based case-control study. Neurology. 1991. pp. 1393–1393. doi:10.1212/wnl.41.9.1393

21. Green RC, Cupples LA, Kurz A, Auerbach S, Go R, Sadovnick D, et al. Depression as a risk factor for Alzheimer disease: the MIRAGE Study. Arch Neurol. 2003;60: 753–759.

22. Ownby RL, Crocco E, Acevedo A, John V, Loewenstein D. Depression and risk for Alzheimer disease: systematic review, meta-analysis, and metaregression analysis. Arch Gen Psychiatry. 2006;63: 530–538.

23. Harder VS, Stuart EA, Anthony JC. Propensity score techniques and the assessment of measured covariate balance to test causal associations in psychological research. Psychol Methods. 2010;15: 234.

24. Heckman JJ, Vytlacil E. Structural Equations, Treatment Effects and Econometric Policy Evaluation. National Bureau of Economic Research; 2005 Apr. Report No.: w11259. doi:10.3386/w11259

25. Wong J, Lewis R, Wardrop M. Efficient Computation of Linear Model Treatment Effects in an Experimentation Platform. 2019. Available: http://arxiv.org/abs/1910.01305
