# Supplementary material for: Counterfactual analysis of differential comorbidity risk factors in Alzheimer’s disease and related dementias
Source: PLOS Digit Health. 2022 Mar 15;1(3):e0000018. doi: 10.1371/journal.pdig.0000018 (PMC9931358; doi:10.1371/journal.pdig.0000018)
Supplement: Attachment — Submitted filename: 2nd Response to Reviewer.pdf [file pdig.0000018.s006.pdf]

(PDIG-D-21-00037R1) Counterfactual Analysis of Differential Comorbidity Risk Factors in Alzheimer's Disease and Related Dementias

Response to Reviewers

We thank the reviewers for the constructive comments. We have significantly modified the manuscript to meet the reviewers' suggestions.

Reviewer 2

> The outcomes that have been provided are still subjective. We expect that definitions, outcomes, and interpretations are all provided clearly when presenting results.

➜ We thank Reviewer 2 for raising this question. We assume Reviewer 2 refers to the outcome related to Supplementary 2 and Supplementary 4 based on the following questions, which is about identifying i) robust edges and ii) distinct edge in two racial groups. We have revised the corresponding section (Page 12) significantly to address Reviewer 2's concern.

> A. In Supplementary 2, how many times must it be detected among the ADRSs to be regarded a meaningful outcome? It is essential to define the criteria or explain the rationale advance.

➜ Thank you for pointing this out. The final causal graph of a racial group is decided by aggregating the results on each data subset using majority voting. Similar method has also been used in other papers, such as

Sinha, Meghamala, Prasad Tadepalli, and Stephen A. Ramsey. "Voting-based integration algorithm improves causal network learning from interventional and observational data: an application to cell signaling network inference." Plos one 16, no. 2 (2021): e0245776.

We added the rationales of using voting-baed methods in the paper:

"The causal structure, however, can vary by input data, which hinders the robustness of the structure. Graph combination methods are usually adopted to increase robustness of the inference results.(Tillman et al. 2008; Claassen and Heskes 2010; Triantafillou and Tsamardinos 2015) We used voting-based causal graph combination to obtain a robust and unbiased estimation of the causal graph. The voting-based method can significantly reduce the false positives and increase the overall robustness of the graph learning.(Sinha et al. 2021) We leveraged the majority voting technique by randomly splitting the entire data into ten

sub-datasets while withholding 10% of the original data each time. We applied the PC algorithm (with a significance level of 0.05) on each of the ten sub-datasets and aggregated the ten results. A directed edge will present in the final ensembled causal graph if it appears in more than half of all the causal graphs. We repeated the same procedure on each racial group and derived the final causal graphs of the two racial groups."

B. In Supplementary 4, A Venn diagram was used to show the differences in node across racial groups. However, a threshold for declaring difference between the two groups must be established in advance.

➜ Thank you for pointing this out. We agree with the reviewer. Indeed, we realize that in fact there is no 'threshold' or 'criteria' on the node's neighbor Venn diagram to decide if the two causal graphs are sufficiently different. (Initially, we added the Venn diagram to provide an overview of the difference between the two causal graphs intuitively. We delete them and only report the standard comparison metrics (SHD, GED) to compare the difference in the paper.) In fact, we want to emphasize that the purpose of this study was to study the causal effect of comorbidities to ADRD, but not to compare the difference between the two causal graphs. In Supplementary document 4, we deleted the Venn diagrams and only kept the two causal graphs.
